# Reconstruction and modeling protein translocation and compartmentalization in *Escherichia coli* at the genome-scale

**DOI:** 10.1186/s12918-014-0110-6

**Published:** 2014-09-18

**Authors:** Joanne K Liu, Edward J O’Brien, Joshua A Lerman, Karsten Zengler, Bernhard O Palsson, Adam M Feist

**Affiliations:** Bioinformatics and Systems Biology, University of California San Diego, 9500 Gilman Drive, Dept. 0419, La Jolla, CA 92093 USA; Department of Bioengineering, University of California San Diego, 9500 Gilman Drive, Mail Code 0412, La Jolla, CA 92093 USA; Department of Pediatrics, University of California San Diego, 9500 Gilman Drive, Mail Code 0412, La Jolla, CA 92093 USA; Novo Nordisk Foundation Center for Biosustainability, Technical University of Denmark, Lyngby, Denmark

**Keywords:** Constraint-based modeling, gene expression, metabolism, protein translocation, compartmentalization

## Abstract

**Background:**

Membranes play a crucial role in cellular functions. Membranes provide a physical barrier, control the trafficking of substances entering and leaving the cell, and are a major determinant of cellular ultra-structure. In addition, components embedded within the membrane participate in cell signaling, energy transduction, and other critical cellular functions. All these processes must share the limited space in the membrane; thus it represents a notable constraint on cellular functions. Membrane- and location-based processes have not yet been reconstructed and explicitly integrated into genome-scale models.

**Results:**

The recent genome-scale model of metabolism and protein expression in *Escherichia coli* (called a ME-model) computes the complete composition of the proteome required to perform whole cell functions. Here we expand the ME-model to include (1) a reconstruction of protein translocation pathways, (2) assignment of all cellular proteins to one of four compartments (cytoplasm, inner membrane, periplasm, and outer membrane) and a translocation pathway, (3) experimentally determined translocase catalytic and porin diffusion rates, and (4) a novel membrane constraint that reflects cell morphology. Comparison of computations performed with this expanded ME-model, named *i*JL1678-ME, against available experimental data reveals that the model accurately describes translocation pathway expression and the functional proteome by compartmentalized mass.

**Conclusion:**

*i*JL1678-ME enables the computation of cellular phenotypes through an integrated computation of proteome composition, abundance, and activity in four cellular compartments (cytoplasm, periplasm, inner and outer membrane). Reconstruction and validation of the model has demonstrated that the *i*JL1678-ME is capable of capturing the functional content of membranes, cellular compartment-specific composition, and that it can be utilized to examine the effect of perturbing an expanded set of network components. *i*JL1678-ME takes a notable step towards the inclusion of cellular ultra-structure in genome-scale models.

**Electronic supplementary material:**

The online version of this article (doi:10.1186/s12918-014-0110-6) contains supplementary material, which is available to authorized users.

## Background

Compartmentalization provided by membranes is essential for life. Compartmentalization allows unique internal microenvironments, permits harvestable energy gradients, provides organizational structure, protects the cell, and more. Membranes also represent significant physical barriers. Thus, cells have evolved pathways that allow molecule transport between compartments. As a gram-negative bacterium, *Escherichia coli* has two membranes: An inner, tightly regulated membrane and an outer, more porous membrane (see [1,2] for review). In order to achieve desired membrane functions, *E. coli* has evolved a system to translocate proteins into their appropriate locations.

There is a wealth of scientific information on protein translocation processes, but holistic studies on their system-wide effects are lacking. Such genome-wide studies are important as protein translocation enables key cellular functions. These functions need to be put into context of all other cellular functions to understand their energetic requirements, general interactions and balance with the rest of the cell. To do so, one must take a systems approach, where comprehensive molecular processes and interactions are reconstructed into a self-consistent and computable format. A couple of recently published studies have taken steps in this direction. In a comprehensive approach to cellular processes, the recent whole-cell model of *Mycoplasma genitalium* incorporates a SecA + Sec translocase pathway into one of its protein formation modules [3]. In this model, translation is uncoupled from translocation, even though the two processes can happen concurrently [4]. Furthermore, protein translocation rates are not calculated *de novo* but are instead based on user-inputted gene expression levels and energy-carrier metabolite concentrations (calculated prior from a separate module). Thus, set expression levels of protein translocases operate as a constraint on other processes; for example, metabolism uptake is dependent on the number of transporters. Additionally, membrane lipid formation is driven by a biomass objective function [3], whereas a computation based on a cell’s surface area might be more appropriate. In another study, a larger effort was focused on the genome-scale reconstruction of the protein secretion pathway in *Saccharomyces cerevisiae* [5]. This model of protein secretion is ‘stand-alone’ and is not integrated with additional cellular processes. It can be used as a scaffold on which omics data (e.g., RNA-seq) can be overlaid to estimate effects of protein abundance and metabolic costs of translocation on the cell. Although these models contain some detail about protein translocation, both are reliant on expression data input and are not dependent on the demands of cellular events. Finally, another notable model incorporated membrane space into a genome-scale model of *E. coli* to demonstrate that while the membrane may cap certain fluxes, leading to simultaneous respiration and fermentation at high growth rates, metabolic demands drive the membrane proteome. Although this model lacks the process of protein translocation and has only four integral proteins, it demonstrated that the consequence of protein translocation, namely compartment formation, truly constrains cellular events [6].

A recent genome-scale model of metabolism and gene-expression of *E. coli*, called a ME-model [7] or specifically, the retroactively named *i*OL1650-ME model (following a previous naming convention [8]), affords us the opportunity to integrate protein translocation seamlessly with cellular processes. Although *i*OL1650-ME describes the synthesis of all the proteins in the proteome, the proteins are not compartmentalized. In this work, we significantly expanded the validated *i*OL1650-ME model [7] to include a comprehensive reconstruction of protein translocation pathways. The expanded *i*OL1650-ME includes a reconstruction of lipoprotein biogenesis, the incorporation of four distinct protein compartments (cytoplasm, periplasm, the inner and the outer membrane), published enzymatic rates of the translocases and diffusion rates of outer membrane porins, and a membrane constraint based on cell morphology all integrated into one reconstruction. The expanded model, hereafter referred to as *i*JL1678-ME, allows for *de novo* prediction of enzyme abundances and their cellular location as well as the constraining effects of membrane production. We apply *i*JL1678-ME to show how it is predictive of compartmentalized cellular content for validation, describe its utility and limitations, and show how it can be applied to examine a broadened scope of applications including targeted inhibition of proteins.

## Results and discussion

All proteins in *E. coli* are synthesized in the cytoplasm, but over 20% of *E. coli*’s protein-coding open reading frame (pORF) are annotated to encode protein with non-cytoplasmic functions, and an estimated 15% of cellular protein mass is in the cell envelope [9,10]. These proteins are assisted by translocase complexes to get to their cellular destinations. Depending on their final location and biochemical properties, the translocation route taken for a particular protein involves one of three integral inner membrane translocases (Sec, Tat, and YidC) and perhaps an outer membrane translocase (LolB and Bam) (see [1,11] for review). The most-studied and ubiquitous translocase is the Sec complex [12]. The channel-forming Sec protein has two chaperone pathways that converge on it. One, the SRP/Sec pathway, brings nascent peptides to the Sec complex and primarily uses the kinetic energy of translation to drive protein integration into the inner membrane [4,13,14]. Sometimes, the mediator YidC binds to Sec complex to enhance proper membrane integration, but on its own, YidC is an insertase that translocates a couple of essential proteins [15-17]. Alternatively, proteins moving to the periplasm and beyond generally follow the SecB/Sec pathway which uses an ATPase, SecA, to thread chaperoned, unfolded proteins through the Sec complex and into the periplasm [18-21]. Furthermore, non-cytoplasmic, folded proteins which often contain cofactors take the Tat translocase, a dynamic protein complex that recruits TatA subunits to adjust its channel size appropriately and is driven by an electrochemical gradient [22-24]. To get to the outer membrane, proteins must first cross the inner membrane, then take one of the two pathways: Lol or Bam. The Lol pathway excises lipoproteins from the inner membrane and incorporate them into the outer membrane [25,26]. In the Bam pathway, unfolded β-barrels are chaperoned in the periplasm, typically by SurA [27,28], to the Bam complex, which facilitates their proper insertion into the outer membrane [29]. Alterations to these pathways exist, but these five translocation pathways are thought of as canonical pathways [25,30]. All this information enables a bottom-up reconstruction of the protein translocation network in *E. coli*.

### Reconstruction of protein translocation processes and their incorporation into *i*OL1650-ME

**Figure 1:**
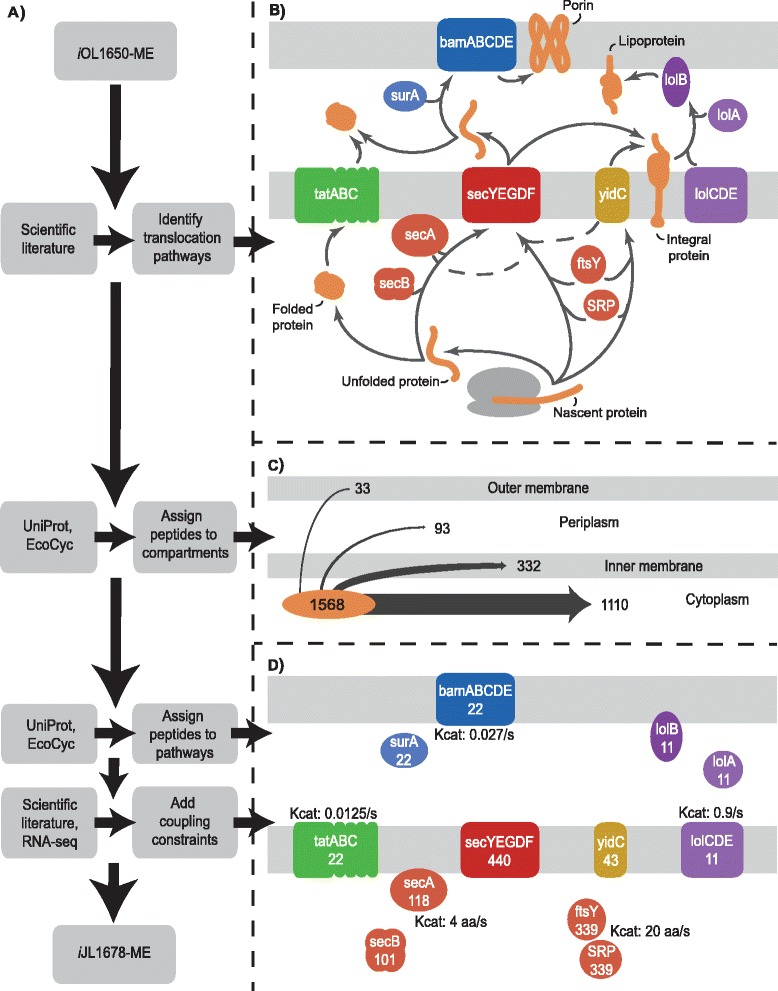
**The workflow utilized and resulting network for reconstructing protein translocation in** ***E. coli.*** **(A)** An outline of the workflow used to reconstruct the protein translocation network in *E. coli*. At each step, various sources of data were used as inputs to the workflow. The resulting general network, compartmentalized content, and pathway breakdown are shown in greater detail to the right. **(B)** A diagram of the translocation pathways included in the reconstruction: SRP/Sec, SecB/Sec, Tat, YidC, Lol, Bam pathways, and three alternatives (dashed lines). Proteins that allow translocation are labeled in white while translocated protein types are labeled in black. Lipoprotein biogenesis is not depicted. **(C)** Model-simulated pORFs were assigned to one of four compartments. The numbers denote how many of the 1,568 proteins will end up in each compartment. **(D)** Each non-cytosolic pORF was assigned to a translocation pathway. Numbers in white are how many pORFs require that translocation-associated protein. The model also underwent several other updates, including the addition of known turnover rates that are denoted by black numbers.

A bottom-up procedure to reconstruct the network of protein translocation and lipoprotein biogenesis within a genome-scale model of metabolism and gene-expression in *E. coli* [7] was developed (Figure 1A). The result of implementing this procedure was a biochemically, genetically, and genomically structured network [31] that enabled the analysis of the molecular effects of protein translocation in context of other networks using constraint-based analysis methods. The network reconstruction procedure involved five major phases.

#### Reconstruction of protein translocation pathways

Through an extensive literature search, the SecB/Sec, SRP/Sec, Tat, Lol, Bam, and YidC insertion translocation pathways were identified for inclusion into the reconstruction (Figure 1B) (see [1,11] for review). Three additional pathways were also included, based on case studies demonstrating that the SRP/Sec pathway occasionally requires assistance from YidC and/or SecA to have properly formed integral proteins [25,30,32]. In addition to protein translocation, lipoprotein biogenesis pathways were reconstructed, as lipoproteins are located in membranes and are essential through their structural and functional uses (Methods & Additional file 1) [33-35]. In the end, 27 pORFs and one RNA gene, which together form 16 protein complexes, were added to the model to enable protein translocation (Additional file 2: Tables S1 and S2). Furthermore, based on the sequence of events in each of these pathways, a set of mechanistic reactions (i.e., template reactions [36]) were developed that could be applied to and individualized for every pORF (Additional file 1).

#### Compartmentalization

The incorporation of protein translocation pathways requires proteins to have defined compartmentalization. First, two new compartments, inner and outer membranes, were added to the three existing compartments in *i*OL1650-ME (cytoplasm, periplasm, and extra-cellular) [7]. Using the protein databases EchoLocation [37], Uniprot [38], and Ecocyc [39] as well as the bioinformatic programs PSORTb [40] and TMHMM [9], the 1,568 pORFs included in the reconstruction were assigned to compartments (Figure 1C). pORFs with a transmembrane component or a lipid membrane anchor were assigned to either the inner or outer membrane; otherwise, pORFs were either cytoplasmic or periplasmic. Proteins composed of multiple pORFs were assigned to the compartment of its components (Additional file 2: Table S2), but if any of its pORFs was in a membrane then the entire complex was assigned to that membrane, with the outer membrane taking precedent over the inner (e.g., AcrAB-TolC multidrug efflux system is assigned to the outer membrane). For example, ATP synthase has pORFs located in the inner membrane (AtpB, AtpC, AtpE, AtpF) and cytoplasm (AtpA, AtpD, AtpG, AtpH), but the synthase itself is assigned to the inner membrane so that it may interact with metabolites in both the cytoplasm and periplasm.

The compartment assignment resulted in 71% of pORFs being assigned to the cytoplasm, 21% to the inner membrane, 6% to the periplasm, and 2% to the outer membrane.

#### Assigning translocated proteins to pathways

Protein translocation reactions were formulated for each pORF. Using a set of rules based on experimental data, protein location, and physical properties (Additional file 2: Table S3), non-cytoplasmic annotated pORFs were assigned to translocation pathways (Figure 1D). The developed template reactions allowed for the methodological creation of each pORF’s translocation reactions and their subsequent incorporation into the reconstruction. Additional pathway development steps included determining the amount of ATP hydrolyzed by SecA for each pORF (i.e., 1 ATP per ~25 amino acids) [41], assigning 23 pORFs to lipoprotein biogenesis [37], and calculating the number of TatA’s needed for each Tat-translocated pORF [23] (Additional file 1, Additional file 2: Table S1, Additional file 3: Figure S1).

Published translocase k_cat_ values were associated with appropriate proteins in the translocation pathways. These values [42-46] were incorporated into the model through coupling constraints [36,47], which account for turnover rates by linking gene expression to metabolism through the dependence of reaction fluxes on enzyme concentration (Figure 1D) [35]. Additionally, outer membrane porins were represented to behave as passive-diffusion channels [2] in the reconstruction. Instead of identical turnover rates for all outer membrane porins in the cell, incorporation of porin-specific coupling constraints allowed the model to account for individualized solute diffusion rates based on effective porin radius, hydrodynamic solute radius, membrane thickness, and growth rate (see Additional file 2: Table S4 for list of solutes, which are also exchange metabolites). This formulation represents the cross-sectional area a solute can pass through and distance a solute had to travel to reach the periplasm [48] (Additional file 1). Without these coupling constraint updates, the model was unable to predict accurate translocase levels (Additional file 3: Figure S2).

#### Incorporating cell-size and membrane constraints

Cell envelope production was fundamentally changed to reflect the cell’s shape and composition more accurately. The previously-developed *i*OL1650-ME accounts for production of kdo 2 lipid IV, phospholipids, and murein through growth rate dependent demands scaled to cell size [7]. These demands were identified as key areas for improvement to a more mechanistic description in *i*JL1678-ME. Changes to the model included adding murein recycling, a lipoprotein demand, and a membrane spatial constraint. The peptidoglycan layer protects the cell from lysis by providing a physical structure, and it also dynamically renews its components by using enzymes located in all compartments of the cell (see [49] for review). To reflect this renewal process, AmpG permease transports anhydro-muropeptides to equal 45% of the murein demand, which causes a murein recycling loop [50]. Lipoproteins are also important for structural integrity, and the number of lipoproteins that have been estimated in a cell, 7×10^5^, is a significant amount of mass [10], so a growth-rate scalable lipoprotein demand, using Braun’s lipoprotein [51], was added. Finally, because there are inner and outer membrane compartments, membrane demands and composition can be more explicitly described with the genome-scale model. Membrane surface area, which is a function of growth rate, is required to be occupied completely by proteins and lipids (see Methods). The surface area of integral proteins was calculated from their mass, except for lipoproteins which were set to the approximate cross-sectional area of their lipid moieties (Additional file 2: Table S5) [10,23,52]. The rest of the outer membrane outer leaflet is filled in with kdo 2 lipid IV while the other three membrane leaflets are occupied by a mixed composition of phospholipids (see Methods for mathematical formulation of the membrane constraint) [53,54]. This novel membrane constraint not only allows a variable membrane proteome, but it also ensures that the cell is completely covered by two membranes.

#### Updating model parameters

Two model parameters were updated to reflect the new reconstruction content. The growth-associated maintenance (GAM) was updated from 35 to 34.98 mmol ATP gDW^−1^ to account for the ATP spent translocating proteins out of the cytoplasm, which is small compared to the cell’s total energy production but expensive per non-cytoplasmic protein (0.02 for translocating 2.3×10-3 mmol protein gDW^−1^, or 85.7 ATP for each non-cytoplasmic protein). Also, the out-of-scope protein proportion of proteome, a parameter introduced in *i*OL1650-ME to account for proteins expressed *in vivo* but not actively utilized by the network reconstruction [7,55], was changed. As *i*JL1678-ME includes more pORFs, this parameter’s value had to be reduced by the expressed mass of new protein content. Thus, the out-of-scope protein proportion was changed from 0.45 to 0.36 to reflect *i*JL1678-ME’s increased comprehensiveness.

Taken in whole, the improved network reconstruction demonstrated that there is enough scientific literature to accurately reconstruct protein translocation in a genome-scale model. As a result of having this reconstruction, it was possible to compute physiological aspects of the cell envelope, which converges to a fully comprehensive *in silico* model of *E. coli* (Additional file 4).

### Proteomic shifts highlight the significance of new content in *i*JL1678-ME

*i*OL1650-ME and *i*JL1678-ME enable quantitative predictions of genome-scale proteome abundances. Instead of requiring input expression data, these models calculate the proteins necessary to maximize growth rate through a metabolism-centered network. However, not only does *i*JL1678-ME contain more reconstructed content, but it also has a reformulated cell envelope representation that requires more membrane production, phospholipid variety, and murein recycling.

**Figure 2:**
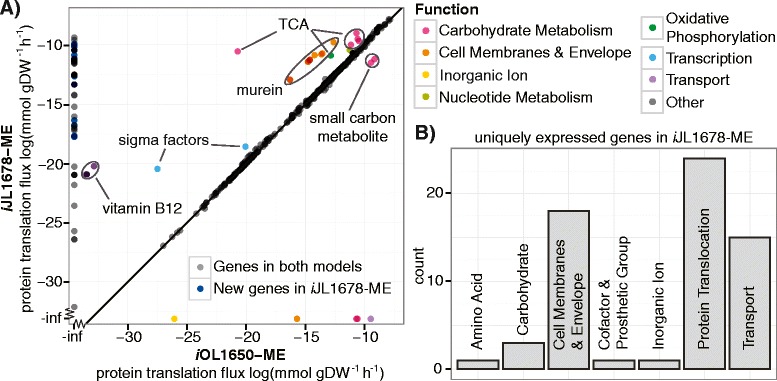
**Proteome expression comparison between** ***i*** **OL1650**-**ME and** ***i*** **JL1678**-**ME.** The difference that the protein translocation reconstruction brings to *i*OL1650-ME is compared through computed protein expression in glucose M9 minimal media conditions. **(A)** Protein translation flux between *i*JL1678-ME and *i*OL1650-ME. The majority of pORF expression (93.5%) are approximately the same in both model simulations, but 4.1% are uniquely expressed in *i*JL1678-ME, and 0.4% is uniquely expressed in *i*OL1650-ME (points along the -inf line). 2.0% of the proteins are differentially expressed, the majority of which are expressed to a greater extent in *i*JL1678-ME than in *i*OL1650-ME, but two proteins involved in small carbon metabolism (EutD and PurT) are expressed lower. **(B)** Histograms detailing the functional annotations of the uniquely expressed genes within the two models.

To demonstrate the difference between the two ME-models, the computed protein expression fluxes in glucose M9 minimal media were compared (Figure 2, *in silico* media composition given in Additional file 2: Table S6). Although the majority of pORFs (1475) were approximately the same in both model simulations, 32 of the genes were differentially expressed, and a number of proteins were uniquely expressed (Figure 2A). Clearly, accommodating protein translocation has a systemic effect on the computed proteome.

Looking first at pORFs expressed in both models, the largest outlying subgroup is the cell membrane and envelope related proteins. This differential expression was due to the addition of murein recycling, which increases overall murein production (145%) and associated ATP expenditure (140%, which is 2.3% of all ATP production in *i*JL1678-ME). It has been previously reported that murein recycling can come as a significant cost to the cell [50]. As for carbohydrate metabolism, the porin coupling constraint forced *i*JL1678-ME to consider the slower diffusion rate of acetate verses gaseous molecules; thus, *i*JL1678-ME utilized acetate overflow (i.e., fermentation) pathways less than *i*OL1650-ME. Not only was its acetate secretion less (1.5 verses 8.1 mmol gDW^−1^ h^−1^), but it also downregulated two genes involved in small carbon molecule metabolism (*eutD* and *purT*). Instead, *i*JL1678-ME adjusted its energy production pathways so that more of its ATP was generated through oxidative phosphorylation. As a consequence, expression of TCA cycle proteins and succinate dehydrogenase was greater. Finally, the collective increase in protein expression due to the expanded scope of *i*JL1678-ME led to greater expression of transcription, vitamin B12 transporters, and nucleotide metabolism proteins.

When examining the uniquely expressed genes, 65 genes were unique to *i*JL1678-ME (Figure 2B), and 6 to *i*OL1650-ME. Of the uniquely expressed pORFs in *i*JL1678-ME, 42% were reconstructed in this paper and thus not contained in *i*OL1650-ME. The rest were due to murein recycling, more phospholipid variety (as part of the membrane constraint), and an increase in oxidative phosphorylation, which in turn required heme metabolism. As for the uniquely expressed proteins in *i*OL1650-ME, these proteins were due to isozymes employed (e.g., AcnA verses AcnB in *i*JL1678-ME).

In summary, the increased scope of modeled genes in *i*JL1678-ME caused a notable change in protein expression levels, and these shifts can be directly attributed to model updates and constraints derived from biochemical knowledge available in literature. The resulting proteomic content was examined further.

### *In silico* computations recapitulate *in vivo* data

To estimate the accuracy of the *i*JL1678-ME *in silico* proteome, glucose M9 minimal media simulation results were compared to experimental data (Additional file 2: Table S6). Unlike *i*OL1650-ME, *i*JL1678-ME calculates a compartment-specific proteome with absolute protein levels. Although this ability may be especially useful in studying the membrane proteome, an area plagued by hardship due to its hydrophobic and amphiphilic nature, it has also created difficulty in comprehensively evaluating *i*JL1678-ME’s results. Even though the correlation between the transcriptome and proteome is poor on a protein-to-transcript level [56,57], RNA-seq is a robust currently-available omic data-source which covers genome-scale expression in all compartments. Assuming that discrepancies in transcript-to-protein ratios are reduced through averaging, RNA-seq data (GEO accessions: GSE48324 [58] and GSE61327 [59]) was assumed as a one-to-one proxy for protein levels. Protein masses were calculated from amino acid sequences and normalized by relative fractional proteome mass. Once a comprehensive quantitative proteomics dataset is available, it will be important to validate that the same functional groups are under-predicted.

**Figure 3:**
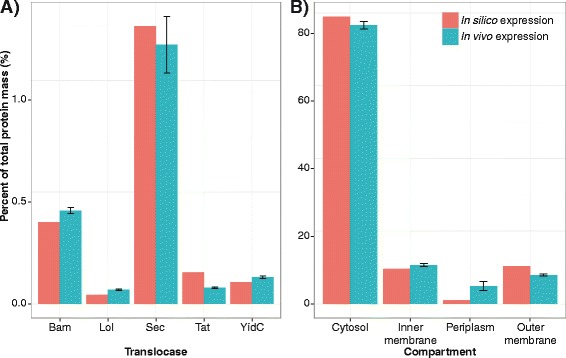
**Comparison of** ***in silico*** **predicted protein masses verses** ***in vivo*** **measurements for reconstructed content specific to** ***i*** **JL1678**-**ME.** Bar graphs showing simulation results (pink) of protein levels from the reconstructed *i*JL1678-ME verses measured *in vivo* expression levels (blue) using averaged RNA-seq as a proxy for protein production. Results were taken from glucose M9 minimal media conditions. **(A)** Translocase protein levels. **(B)** Percentage of protein mass in each of the four compartments.

Since the network reconstruction expanded the scope of *i*OL1650-ME, we sought to validate the new features of the genome-scale model. The computed mass of all proteins associated with a translocation pathway (color labeled in Figure 1B) as a fraction of total cellular protein mass is largely similar to *in vivo* data (Figure 3A, Additional file 3: Figure S3). The most notable outlier is the Tat pathway. The difference between *in silico* and *in vivo* expression may be due to the fact that a TatBC complex forms multiple channels to simultaneously translocate substrates [60,61], but in *i*JL1678-ME model, each TatBC complex translocates a single substrate at any point in time. To explore the possibility of a different representation for TatBC, the mass of TatBC was adjusted by four-fold (the maximum demonstrated number of bound precursor proteins) and this improved the *in vivo* to *in silico* correlation (R^2^ = 0.897 to 0.925, p-value = 0.014 to 0.009), which hints at the possibility TatBC commonly forms multiple channels per complex *in vivo*. These results demonstrate that bottom-up reconstruction approaches and constraint-based modeling can estimate relative protein levels when incorporated with turnover rates and metabolic demands and serves as validation of the reconstructed content (see Additional file 3: Figure S2 for translocation without k_cat_).

*i*JL1678-ME’s ability to accurately compute protein amounts extends to compartmentalization, which is enabled due to protein translocation (Figure 3B). Simulation results predict that the mass of cytoplasmic proteins constitute approximately 79% of the proteome, while the inner membrane protein masses are 10%, periplasmic 1.0%, and outer membrane 10%. Calculating these same values for *in vivo* measurements gave 76.6%, 10.6%, 4.9%, and 7.9%, respectively. In a complementary analysis, *i*JL1678-ME estimated outer membrane protein values closer to published numbers than *in vivo* (RNA-seq) data’s approximation of the outer membrane proteome. The *in silico* protein numbers reflect experimental published amounts at 7.2×10^5^ lipoproteins verses 7×10^5^ and 1.5×10^5^ porins verses 2×10^5^ [10], which implies that the RNA-to-protein ratio is not one-to-one for outer membrane proteins. As there are less proteins in the non-cytosolic compartments, the averaging effect of large groups is less effective, which may explain the discrepancy.

**Figure 4:**
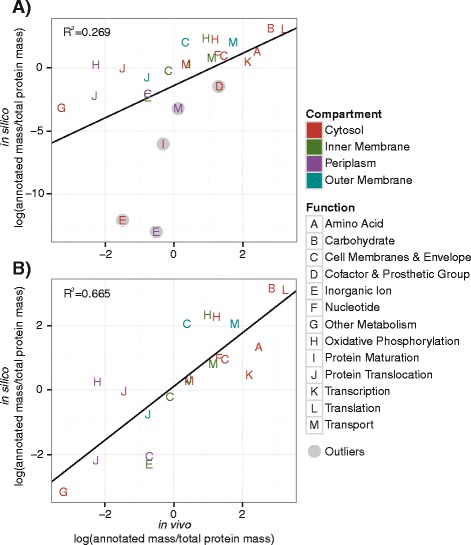
**Analysis of** ***in silico*** **predicted protein masses verses** ***in vivo*** **measurements.** Predicted (*in silico*) versus measured (*in vivo*) protein masses that were reconstructed in *i*JL1678-ME were categorized by function and compartment. Subgroups with zero values were removed from further calculations. **(A)** The linear model between *in silico* and *in vivo* protein mass predictions (p-value = 6.6x10^−3^). The outliers had standardized residues that fell outside of the normal distribution curve as formed by the other points (Additional file 2: Figure S4). **(B)** The outliers were removed, and the linear model between *in silico* and *in vivo* protein mass predictions was recalculated (p-value = 6.6x10^−6^).

Where do the similarities and differences between the computed and measured compartment-specific protein mass arise from? To answer this question, the protein masses were broken down into smaller subgroups, as labeled in *i*JO1366 which used EcoCyc and GO annotations [39,62]. All 1,568 pORFs were categorized by functional annotation as opposed to a gene-by-gene comparison, with the assumption that a larger sample size would reduce the discrepancies between protein and RNA abundances. A comparison between computational predictions and experimental data was performed using linear regression of log-log values with zero values being removed from further calculations (Figure 4). A normal probability plot of the standardized residuals of the initial model (Additional file 3: Figure S4) revealed that while most points could be described by a normal distribution, five points describing lowly-expressed functions in *i*JL1678-ME were out of range (Figure 4A). These five points were separated for further analysis while the reduced set of points was refitted, resulting in a more accurate linear model (Figure 4B).

Due to their departure from normalcy, the five outliers in Figure 4A were examined to identify reasons for modeled discrepancies. The five points covered genes involved with inorganic ions, cofactor and prosthetic groups, protein maturation, and metabolite transportation. Not only is the available knowledge of metal ion and cofactor requirements sparse [63], but the model demands the incorporation of only the most necessary groups into proteins. As result, expression of inorganic ion, cofactor, and prosthetic related pORFs are low. Similarly, protein maturation pORFs are required for proper inclusion of ions and groups; they also assist mis-folded proteins, whose possibility are not computed in optimal situations. Lastly, *i*JL1678-ME predicts a lower periplasmic mass for small metabolite transportation as compared to *in vivo* data. Closer examination of this functional group revealed that the model has severely decreased the diversity of ABC transporters to five protein species. However, *E. coli* produces multiple species of ABC transporters in preparation for environmental changes [64]. This readiness to consume a variety of substrates improves the cell’s overall fitness, but when confronted with glucose as the sole carbon substrate, the varied over-expression limited the predicted optimal growth rate, according to *i*JL1678-ME.

### Applications predict the effect of molecular perturbations

Genome-scale models of metabolism have enjoyed many successes in elucidating interactions, metabolic engineering, drug targeting, and more. Up to this point in time, perturbations in genome-scale models are often focused on gene knockouts and constraining a particular reaction to a bound [65]. *i*JL1678-ME can be used to provide new insights which cannot be currently be achieved with existing models; that is, *i*JL1678-ME can be used to estimate the detailed effects of molecular processes and physical parameters and on a much broader scale. This ability of *i*JL1678-ME will be demonstrated through two examples: Membrane crowding and Sec pathway inhibition.

#### Assessing the consequences of membrane crowding

Molecular crowding in the finite space of cells limits metabolic activity [6,66]. Such crowding constraints are found both in the volume of the cell (also called ‘packing’ constraints) as well as the surface area of its membranes. *i*OL1650-ME, and consequently *i*JL1678-ME, implicitly considers volume crowding effects because density is constrained based on the overall growth rate [7]. Limited surface area in the membranes are thought to constrain major aspects of metabolism and physiology; for example, it may force *E. coli* to employ a mixture of respiration and fermentation to maximize growth rate [6,67]. Thus, as part of the reconstruction process, a constraint on the fraction of protein in the membranes was incorporated into *i*JL1678-ME (Additional file 1). This membrane constraint is mechanistic and imposed on a genome-scale, thereby representing a unique opportunity for a detailed assessment of the consequences of limited membrane space. The results of restricting the total surface area of integral membrane proteins in the model are described.

**Figure 5:**
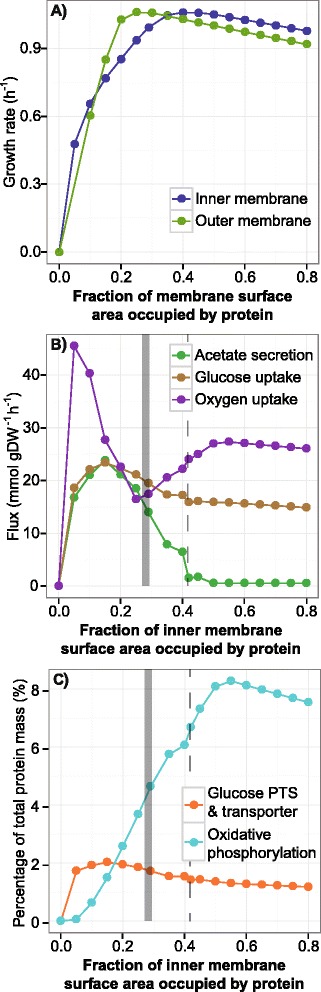
**The effects of constraining the amount of membrane surface area that may be occupied by protein.** Shown here is a scatterplot comparing the effects of controlled protein occupancy in the membranes. **(A)** The effects of constraining the protein surface area in the inner and outer membranes. The apex of growth rate occurs at 0.42 fractional area for protein occupancy for the inner membrane and 0.25 for the outer membrane. The growth rate decreases more rapidly if membranes protein were under-produced verses over-produced. **(B)** Acetate secretion, glucose uptake, and oxygen uptake fluxes when constraining inner membrane protein surface area. The gray solid bar represents the RNA-seq derived *in vivo* surface area (+/− one standard deviation), and the dashed line represents the optimal inner membrane surface area occupancy. **(C)** Mass of the electron transport system complexes and glucose transporters when constraining inner membrane protein surface area.

Computations of growth optimization were performed with constraints on the protein-to-lipid surface area ratios in both the inner and outer membranes. These computations revealed that the maximum growth rate was achieved when the fraction of membrane surface area occupied by protein was 42% and 25% for the inner membrane and outer membrane, respectively. Furthermore, over- and under- production of membrane proteins did not affect the maximum growth rate with the same severity. The uneven slopes from the apex at 42% and 25% indicates that over-expression of membrane proteins may be less taxing on growth rate than under-expression, suggesting that it may in the cell’s favor to over-produce membrane proteins than under-produce (Figure 5A).

As the inner membrane contains a diverse set of proteins that are important for metabolism, *i*JL1678-ME was used to examine the effects of spatial limitations on the inner membrane proteome. Although oxidative phosphorylation is much more efficient than alternate energy producing pathways, *E. coli* at high growth-rates and in excess glucose also employs fermentation pathways [68]. The electron transport system (ETS) is embedded in the membrane, and limited membrane space for the ETS may be why *E. coli* resorts to the mixed energy-production strategy [6]. *i*OL1650-ME, on the other hand, predicted that such a phenomenon occurs based on the trade-off between ATP generation and protein production costs [7].

In *i*JL1678-ME, acetate secretion has been almost eliminated compared to *i*OL1650-ME (8.1 to 1.5 mmol gDW^−1^ h^−1^), due to the porin constraint. Differences in diffusion rates for each metabolite allowed the model to recognize that gases diffuse faster than solubilized carbon molecules, and complete metabolism of a carbon source becomes a better investment. However, fermentation returned when the inner membrane protein surface area decreased below 50%, as demonstrated by the increased secretion of acetate (Figure 5B). Within these regions of constraining protein-occupied surface area, the cell model produced less oxidative phosphorylation products, which includes the ETS, instead of glucose PTS permeases and transporters for continued and increased glucose uptake, as previously hypothesized (Figure 5B & C) [6]. At extremely low surface areas allocated to proteins (≤10%), there was not enough room to accommodate NADH dehydrogenase in the membrane. Instead, alternate dehydrogenases were expressed. Thus, to maximize growth rate, *i*JL1678-ME choses to increase fermentation rates with decreased membrane space.

Once membrane space permits complete metabolism of glucose influx at ~50% protein-occupied surface area, fermentation pathways are no longer heavily employed which improves metabolic efficiency, hence the drop in in glucose uptake and increase oxygen uptake (Figure 5B). However, beyond 50%, *i*JL1678-ME makes a trade-off between producing more ETS, an expensive investment, to alternative proteins (Figure 5C). This shift in protein expression to accommodate the trade-off of ETS may play out similarly for proteins not required for metabolism, protein translocation, or metabolite transport but are essential for other processes (e.g., expression of flagella for locomotion).

Where do *in vivo* cells fall along this scan across inner membrane occupancy? The calculated *in vivo* surface area of 28.5%, based on RNA-seq data (Additional file 1), puts a cell below optimal membrane occupancy. Within this range of *in vivo* surface area, the increased acetate secretion hints that membrane space constraints may indeed be why cells employ combinatorial energy production pathways at maximum growth rates, as Zhuang *et al*. had hypothesized [6]. Furthermore, oxygen uptake drops severely when the protein surface area approaches the *in vivo* value of 28.5% (17 mmol gDW^−1^ h^−1^ which is close to the measured values of 15 mmol gDW^−1^ h^−1^ [69] and 18 mmol gDW^−1^ h^−1^ [70]). This finding implies that a finite inner membrane protein surface area can limit the oxygen uptake and usage rate, thereby lowering the growth rate to less than the maximum potential.

#### Perturbations in network performance by changing enzymatic efficiency

The Sec pathway is a key pharmaceutical target due to its ubiquity and essentiality. For example, SecA is particularly attractive since it does not have a human homologue, and a recent non-cellular assay for SecA activity was developed specifically for drug discovery [71]. However, effects of decreased Sec translocase activity on a cell are largely unknown. While reactions in metabolic models can be capped to mimic protein inhibition, *i*JL1678-ME takes this ability further by targeting enzymatic efficiencies, similar to the effects of drugs. Thus, the impact of inhibiting Sec translocation on overall cellular phenotype was analyzed with *i*JL1678-ME by targeting key enzymes. SecA is the energy driver for the SecB/Sec pathway, and the ribosome is the energy driver for the SRP pathway. Together, these two pathways meet at SecYEGDF (Figure 1B). Due to their importance, these three proteins were inhibited.

**Figure 6:**
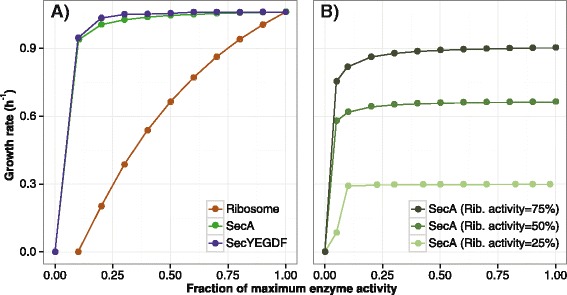
**The effects of inhibiting SecA on growth rate. (A)** A scatterplot showing the effects of decreasing enzyme efficiency of several key enzymes involved in Sec translocation (ATPase SecA, the channel SecYEGDF, and ribosome) have on growth rate. The growth rate was predicted by decreasing turnover rate (i.e., k_cat_) of SecA, SecYEGDF, and ribosome and optimizing for growth rate. Simulations were performed with an upper limit of 0.5 of the membrane protein surface area occupancy. **(B)** The effects of simultaneously inhibiting SecA and ribosome.

When the k_cat_ values of SecA, SecYEGDF, and the ribosome were reduced in a step-wise manner, growth rate was affected differently in each situation (Figure 6A). The relationship between ribosome inhibition and growth rate is nearly linear. SecA and SecYEGDF, on the other hand, behave in a hyperbolic manner. Thus, unlike ribosome, the activity of SecA or SecYEGDF must be nearly eliminated (i.e., SecA < 2.5%, SecYEGDF < 5%) to reduce the growth rate by half. A closer look at these extremely low enzymatic rates reveals that the *in silico* membrane proteome was dominated by SecYEGDF. Therefore, membrane occupancy was capped at 50%, as done by Zhuang *et al*. [6], to determine whether spatial limitations may change the overall behavior to Sec pathway perturbations. The inhibition simulations were repeated, showing that ribosome was not affected by membrane limitations, while effects were observed when SecA and SecYEGDF’s turnover rates dropped below two amino acids per second (Additional file 3: Figure S5). However, regardless of membrane space, both SecA and SecYEGDF must be severely inhibited to significantly decrease growth rate. This example of targeting Sec translocation shows that *i*JL1678-ME can be used to discover cellular effects of selected perturbations. Other molecular behaviors, like combinatorial drug effects, may find similar answers through *i*JL1678-ME. For example, simultaneously targeting the two chaperone pathways for SecYEGDF, namely SecA and ribosome, is not a synergistic approach, and SecA must still be targeted for complete inhibition to significantly lower the growth rate (Figure 6B).

## Conclusions

Taken in whole, *i*JL1678-ME stoichiometrically represents the wealth of knowledge known for protein translocation of *E. coli* in an integrated and computable format. For the first time, a bottom-up stoichiometric reconstruction (with turnover rates) predicted protein levels without expression data as inputs and imposed constraints. Furthermore, the ability to explicitly model protein translocation and compartmentalization of proteins is a significant advancement for genome-scale models, as it alleviates the need for fixed demands for the newly reconstructed content. In combination with the membrane constraint, proteomic predictions represent a milestone for constraint-based modeling. As an example, *i*JL1678-ME could be utilized for designing fine-tuned engineered strains by identifying how the membrane proteome may react to overexpression of non-cytoplasmic proteins and for determining ways to counteract undesired effects through selective gene manipulation. Through exploration of modeled membrane formation contextualized within protein translocation and metabolism, *i*JL1678-ME demonstrated that bottom-up systems-biology can be used to predict and analyze cellular physiology, thereby providing an opportunity to assist and supplement research on fundamentally challenging areas which may otherwise be difficult to study.

Improvements in *i*JL1678-ME are likely to come through further experimental evidence. For example, more elucidation is required on the exact stoichiometry of TatA proteins per substrate and complex before such information can be incorporated into *i*JL1678-ME. Other ME-model based reconstructions may include a module to simulate plasmid induction and subsequent protein secretion. Finally, *i*JL1678-ME’s predictive capabilities could be improved by incorporating data types such as ribosome profiling, quantitative proteomics, and additional k_cat_ values. In conclusion, ME-models with compartmentalization and membrane constraints open exciting new avenues for the use of genome-scale models to interpret biological functions, to form the basis for strain designs, and understand infectious disease.

## Methods

### Reconstruction

A metabolism and gene expression model of *E. coli*, retroactively named here *i*OL1650-ME following an established convention [8], was used as the starting basis on which protein translocation reconstruction was built upon [7].

Literature review led to identification of five main translocation pathways plus three alternate assisting proteins. These pathways were developed into template reactions to which each of *i*JL1678-ME’s pORFs could be applied to (Additional file 1).

Based on subcellular location annotations in Echolocation, EcoCyc and Uniprot (discrepancies and unknowns settled through PSORTb and TMHMM), all pORFs and protein complexes were assigned to one of four compartments: Cytosol, inner membrane, periplasm, and outer membrane [9,37-40]. The inner and outer membrane compartments are new additions to *i*OL1650-ME. New genes were also added to allow protein translocation and lipoprotein biogenesis. Reactions in *i*OL1650-ME were modified so that all proteins are compartmentalized. Furthermore, reactions were curated to ensure that reactions account for physical barrier membranes present. For example, if a reaction involves metabolites located in the cytoplasm and the periplasm, an inner membrane protein must be present for the reaction to occur.

Proteins with known experimental evidence were assigned to their respective translocase pathways. Based on these known peptides and current hypotheses, a set of rules was developed so that proteins without an experimentally-validated pathway could be assigned to one. These rules were established primarily by annotated subcellular location and secondarily by the type of protein (Additional file 2: Table S1). However, each pathway operates at its own speed. *i*OL1650-ME’s coupling constraints offer a solution for this problem, as the coupling constraints put limits on fluxes by linking reactions to enzyme degradation and the catalytic rate k_cat_ (see Additional file 1 for basic formulation and example) [7]. Using this established constraint, turnover rates were applied to the translocase pathways to improve the model’s ability to predict the membrane proteome (see Additional file 3: Figure S2 for translocation without k_cat_). Key proteins of each pathway had calculated turnover rates, and these k_cat_ values were applied to all other enzymes in the pathway that have an interaction with that enzyme. The turnover rates of SecA, LolCDE, Bam, and Tat were all known from literature while the turnover rate for the SRP pathway was assumed to be equal to ribosome translation because of co-translational translocation [42-46]. For Tat-translocated proteins, a best fit polynomial equation for the number of TatA’s verses average channel diameter was used to calculate the number of TatA’s required for each [23]. Protein diameter was calculated by multiplying molecular weight by 1.21 to get volume and assuming a sphere shape [52]. Values were rounded up to the nearest integer.

Lipoprotein biogenesis was also determined to be relevant, and thus was included in the reconstruction process. The model has the flexibility to choose fatty acids from any available phospholipid. The proteins are modified by Lgt, Lsp, and Lnt to become lipoproteins (Additional file 1).

Murein demand was adapted from the original *i*OL1650-ME model. However, since it is known that 45% of murein is recycled, the model is forced to utilize the muropeptide transporter (AmpG), which has been implicated in the process of murein recycling [50], so that the flux of transported murein peptides is 45% of the murein demand (0.01389 mmol gDW^−1^).

### Outer membrane porins

As many as 2×10^5^ porins have been determined to be in the outer membrane [10]. Thus, to accurately account for these pathways, the outer membrane porins were coupled with diffusion rates [48,72,73]. In *i*JL1678-ME, the k_cat_ values of the outer membrane porins are individualized for every combination of solute and porin, producing unique reactions reflecting effective diffusion rates based on diameters of solute and porin (Additional file 1, Additional file 2: Table S4). To calculate the concentration difference between the extra-cellular environment and the periplasm, only porins with calculated effective diameters remained in the model (Additional file 1). The diameters for all possible solutes were calculated using MarvinSketch assuming (1) the solutes were suspended in water (solvent radius: 1.4 Å) and (2) the solvent accessible surface area was a sphere, MarvinSketch 6.1.0, 2013, ChemAxon (http://www.chemaxon.com). With all the values known and inputted, this leaves the concentration difference between the extracellular (C_e_) and periplasm (C_p_), C_e_-C_p_, as the sole variable. Using an initial batch culture simulation in glucose M9 minimal media with the assumption C_p_ < < C_e_, the total flux of metabolite passage through outer membrane porins was calculated. Using *i*JL1678-ME’s flux results of outer membrane trafficking, the known number of porins (2×10^5^ per cell) [10], the solute diffusion rate through porins, and the porin constrain equations (Additional file 1), a series of simulations with varying total solute concentration differences were run to estimate the approximate difference to such that number of porins produced equals the experimental value [74]. This concentration difference, 6.5×10^−4^ was incorporated into the porin diffusion rates as the default value, which may be adjusted by the user.

### Updating parameters

In order to determine how much more cellular mass *i*JL1678-ME explicitly accounts for, RNA-seq was first assumed to be a one-to-one proxy for protein expression levels, and in this dataset, the new pORFs and outer membrane proteins summed to 9.5% of all proteomic mass. As a comparison point, the outer membrane protein mass, i.e. lipoproteins and porins, was experimentally derived to be 7.4% of total proteomic mass [10]. Supplementing 7.4% with the estimated mass of protein translocases and lipoprotein biogenesis proteins from RNA-seq (as there were no experimental protein estimates available in literature) summed to 9.2% of total proteomic mass, which is similar to 9.5%.

The GAM (growth associated maintenance) was updated to account for the amount of ATP used in protein translocation. The ATP flux used in protein translocation by SecA and LolCDE was calculated and subtracted from the GAM value established in *i*OL1650-ME, reducing it from 35 to 34.98.

### Membrane constraints

The combined surface area (SA) of membrane proteins, phospholipids (PE is phosphatidylethanolamine, PG is phosphatidylglycerol, and CLPN is cardiolipin), and lipopolysaccharides (LPS) must equal the total surface area of a cell (equation 1) times four membrane leaflets (equation 2) [54,75]. The surface area of each membrane molecule was determined by its classification (Additional file 2: Table S5). If the molecule was a protein, the protein was assumed to extend through the lipid bilayer and occupy twice the amount of calculated surface area. An additional constraint was imposed so that phospholipid composition would better reflect the diversity of known membranes (equation 3).

1 $$ SA\left(\mu \right)=0.456\pi *{2}^{\frac{\upmu * \ln (2)}{3}}*\left(3.9*{2}^{\mu *\frac{ \ln (2)}{3}}-0.456*{2}^{\frac{\upmu * \ln (2)}{3}}\right)+{\left(0.912\pi *{2}^{\frac{\upmu * \ln (2)}{3}}\right)}^2 $$

2 $$ 4*SA\left(\mu \right)={\displaystyle \sum_{i\ \in\ proteins}}SA\  of\  membrane\_ protei{n}_i+{\displaystyle \sum }SA\  of\ LPS+{\displaystyle \sum }SA\  of\  phospholipids $$

3 $$ {\displaystyle \sum }SA\  of\  phospholipids=77\%*{\displaystyle \sum }S{A}_{PE}+18\%*{\displaystyle \sum }S{A}_{PG}+5\%*{\displaystyle \sum }S{A}_{CLPN} $$

Additional constraints in the *i*JL1678-ME include a variable maximum cap on protein surface area and the option to force the model to produce nonfunctional membrane protein.

This cell envelope demand for LPS and lipids originally appearing in *i*OL1650-ME was removed, which makes the production of these two types of molecules a function of growth rate, protein production, and membrane size. Membrane size was taken to growth-rate dependent as formulated by O’Brien *et al*. (see [7] supplemental materials).

### Analyzing the model

The model was run using batch simulations, as described by O’Brien *et al*. using resources of the National Energy Research Scientific Computing Center, which is supported by the Office of Science of the U.S. Department of Energy under Contract No. DE-AC0205CH11231 [7]. For all analyses performed, the *in silico* media composition was M9 and an excess of glucose (4 g L^−1^) (Additional file 2: Table S6).

Since membrane proteomics is difficult to study; it is even more difficult to obtain absolute numbers comparing relative ratios of protein amounts. Therefore, RNA-seq was used as an *in vivo* proxy for comparison (GEO accessions: GSE48324 [58] and GSE61327 [59]). A 1:1 ratio of protein expression levels to RNA-seq levels (FPKM normalized to overall expression) was assumed. Mass was calculated based on the atomic mass of the primary protein structure multiplied by the flux of protein being produced. In comparing *in vivo* data to *in silico* data, mass was summed up by compartment location, functional annotation, or both (Additional file 2: Table S2). Error bars are 1 standard deviation from two RNAseq runs.

The mass of compartmentalized functional annotations between *in vivo* and *in silico* data was compared on a log-log basis. A simple linear regression model was calculated between the two datasets. The standardized residuals (residual i / standard deviation of residual i) of the *in silico* data was plotted against a rankit score (expected values of the order statistics if the sample is normally distributed), creating a normal probability plot. A line passing through the first and third quartiles revealed points that deviated from a normal distribution (i.e. deviated from the quartile line). These points were removed from the dataset for further analysis and the simple linear regression model was recalculated for the reduced dataset.

### Protein inhibition

To adjust the turnover rate of SecA, the coupling constraint was modified so that it would reflect numbers lower than the published value of 4.0 s^−1^ [43]. Similarly, all coupling constraints involved with SecYEGDF or ribosome were multiple by fractions to lower enzyme efficiencies. To limit membrane inner membrane protein surface area, the variable maximum cap (included as part of the membrane constraint formulation) was set to 0.5.

### Ethics

This research did not involve human subjects, human material, human data, animals, or plants.

## Additional files

Additional file 1
**Supplemental Methods.** In-depth details describing the methodology behind the reconstruction process. Additional file 2
**Supplemental Tables.** Tables describing the changes and numbers used during reconstruction compiled into a single excel file. The table of contents is listed on the first sheet. Additional file 3
**Supplemental Figures.** Supplementary figures and legends describing background analysis to accompany the figures in the paper. Additional file 4
***i*** **JL1678-ME.** The *i*JL1678-ME model in python pickle format along with sample python scripts and a README file to run a simulation.

## Abbreviations

*i*OL1650-ME: A genome-scale metabolic and gene expression model of *E. coli*
*i*JL1678-ME: A genome-scale metabolic and gene expression model of *E. coli* including compartmentalization and protein translocation based of the iOL1650-ME model
pORF: Protein-coding open reading frame
