# Supplementary material for: Reconstruction and modeling protein translocation and compartmentalization in Escherichia coli at the genome-scale
Source: BMC Syst Biol. 2014 Sep 18;8:110. doi: 10.1186/s12918-014-0110-6 (PMC4177180; doi:10.1186/s12918-014-0110-6)
Supplement: Additional file 1 — Supplemental Methods. In-depth details describing the methodology behind the reconstruction process. [file 12918_2014_110_MOESM1_ESM.docx]

Table of Contents

**Reaction templates2**

**Diffusion rates through outer membrane porins3**

**Coupling constraints4**

**Outer membrane porin effective diameters5**

***In vivo* calculations5**

**Functions used in R script for linear analysis5**

**Simplified templates for translocation pathways**

SRP/Sec pathway

Ribosome-nascent_chain + SRP + GTP + FtsY-GTP + Sec_complex 🡪

Ribosome-nascent_chain-Sec_complex (translation) + SRP + FtsY + 2GDP + 2Pi 🡪

Protein (inner membrane) + Ribosome + Sec_complex

SecB/Sec pathway

Peptide (cytosol) + SecB + SecA +Sec_complex + 1 ATP/25 aa 🡪 Peptide (periplasm) + SecB+ SecA + Sec_complex + (1ADP + 1Pi)/25 aa

YidC insertion

Ribosome-nascent_chain + SRP + YidC 🡪 Protein (inner membrane) + Ribosome + SRP + YidC

Tat pathway

Peptide (cytosol) + Tat_chaperone + TatBC + (1+)TatA 🡪 Protein (cytosol) + TatBC + (1+)TatA

Lol pathway

Lipoprotein (inner membrane) + LolCDE + LolA + ATP 🡪

Lipoprotein-LolA (periplasm) + LolCDE + ADP + Pi + LolB 🡪

Lipoprotein (outer membrane) + LolB + LolA

Bam pathway

Peptide (periplasm) + SurA + Bam_complex 🡪

Peptide (outer membrane) + SurA + Bam_complex

**Simplified template reactions for lipoprotein biogenesis**

Peptide (inner membrane) + Lgt + pg 🡪

Prolipoprotein + g3p + Lgt + LspA 🡪

Apolipoprotein + LspA+ amide_linked_fatty_acid + Lnt 🡪 Lipoprotein + apg + Lnt

**Formulation for diffusion rates through outer membrane porins**

*P* Permeability coefficient

*D* Free diffusion coefficient

*d* Thickness of the membrane

*a_o_* total cross-sectional areas of all pores

*A* Total area of the outer membrane

*V* Rate of diffusion of solutes across the outer membrane

*C_e_* Concentration of the extra-cellular solutes

*C_p_* Concentration of periplasmic solutes

*R* Gas constant

*T* Temperature

*N_A_* Avogadro’s number

*n* Dynamic viscosity

*r* Radius of Solute

*R* Radius of pore

*g* Growth rate

Theoretical permeability coefficient

$$P_{theory}=\frac{D}{d}*\frac{a_{o}}{A}*\frac{a}{a_{o}}$$

Ficks first law

$$V=P*A*\left( C_{e}-C_{p} \right)$$

Renkin equation

$$\frac{a}{a_{o}}=\left( 1-\frac{r}{R} \right)^{2}*\left( 1-2.104*\frac{r}{R}+2.09*\left( \frac{r}{R} \right)^{3}-0.95*\left( \frac{r}{R} \right)^{5} \right)$$

Stokes-Einstein

$$D=\frac{R*T}{N_{A}*6*n*\pi*r}$$

Flux of solute through porins of type i

$$V_{solute}=\frac{V_{i}*T*R^{3}*\left( 1-2.104*\frac{r}{R}+2.09*\left( \frac{r}{R} \right)^{3}-0.95*\left( \frac{r}{R} \right)^{5} \right)*\left( C_{e}-C_{p} \right)}{6*n*r*d*g*1200}$$

**Coupling constraints**

Basic formulation

*dil* dilution of enzyme (mmol Enzyme gDW^-1^ h^-1^)

*f* flux through a reaction involving an enzyme (mmol gDW^-1^ s^-1^)

*K_cat_* K_cat_ of an enzyme (s^-1^)

[*E*] enzyme concentration (mmol Enzyme gDW^-1^)

*μ* growth rate (h^-1^)

$$f\leq K_{cat}*[E]$$

$$dil=\mu*[E]$$

$$f\leq\frac{dil*K_{cat}}{\mu}$$

Example coupling constraint

As an example, we will use BamA as it crosses the inner membrane through the SecB/Sec pathway in batch glucose M9 minimal media (as simulated for figures 2-4). This protein is 810 amino acids long, which means that 33 ATP molcules per BamA are required.

From the SecB/Sec pathway template reaction:

BamA[c] + SecB + SecA +Sec_complex + 33 ATP $\underset{\to}{f}$ BamA[p] + SecB+ SecA + Sec_complex + 33 ADP + 33 Pi

Essentially, the flux of BamA being translocated (*f*) must be equal to or less than the total flux of Sec production multiplied by translocation rate at any given moment within the cell. The number of BamA’s required for the simulated situation is 1700 copies, which equals a flux of 2.5 e-9 mmol gDW^-1^ s^-1^. Likewise, the flux production (which is equal to dilution flux in this case) of SecA is 3.07 e-5 mmol gDW^-1^ h^-1^. The final two numbers are the translocation rate of BamA (202.5 s^-1^) and growth rate (1.06 h^-1^).

$$f=2.5*{10}^{-9}\mathrm{mmol}\mathrm{BamA}\mathrm{gDW}^{-1} s^{-1}\leq\frac{3.07*{10}^{-5}*202.5}{1.06}=5.86*{10}^{-3}\mathrm{mmol}\mathrm{SecA}\mathrm{gDW}^{-1} s^{-1}$$

Thus, of all the translocation reactions SecA was catalyzing within a cell in a given second, 4.2e-5% of these reactions are translocating a BamA protein.

**Outer membrane porin effective diameters**

| **Porin** | **Effective diameter** | **Reference** |
| --- | --- | --- |
| ompA | 1.0 nm | Sugawara and Nikaido 1991 |
| ompC | 0.54 nm | Nikaido and Rosenberg 1982 |
| ompF | 0.58 nm | Nikaido and Rosenberg 1982 |

***In vivo* calculations**

$${PORF_{mass}}_{i}=\frac{\left( {Gene\_expression}_{i}*{PORF_{MW}}_{i} \right)}{\sum_{i\in PORF} \left( {Gene\_expression}_{i}*{PORF_{MW}}_{i} \right)}$$

$$Surface\_area=\frac{\sum_{j\in IM\_PORF} {PORF_{mass}}_{j}}{\sum_{i\in PORF} {PORF_{mass}}_{i}}*\frac{1.21*2}{membrane\_thickness}*\frac{in\_silico\_PORF_{mass}}{in\_silic{o\_PORF}_{surface area}}$$

**Functions used in R script for linear analysis**

| **Function** | **Description** |
| --- | --- |
| log() | Computes natural logarithm |
| lm() | Fit linear model |
| rstandard() | Computes standardized residual |
| ggplot() | Plotting system for R |
| qqnorm() | Plots normal QQ plot |
| qqline() | Adds a line through the first and third quartiles on QQ plots |
