# Supplementary material for: Reconstruction and modeling protein translocation and compartmentalization in Escherichia coli at the genome-scale
Source: BMC Syst Biol. 2014 Sep 18;8:110. doi: 10.1186/s12918-014-0110-6 (PMC4177180; doi:10.1186/s12918-014-0110-6)
Supplement: Additional file 3 — Supplemental Figures. Supplementary figures and legends describing background analysis to accompany the figures in the paper. [file 12918_2014_110_MOESM3_ESM.docx]

**
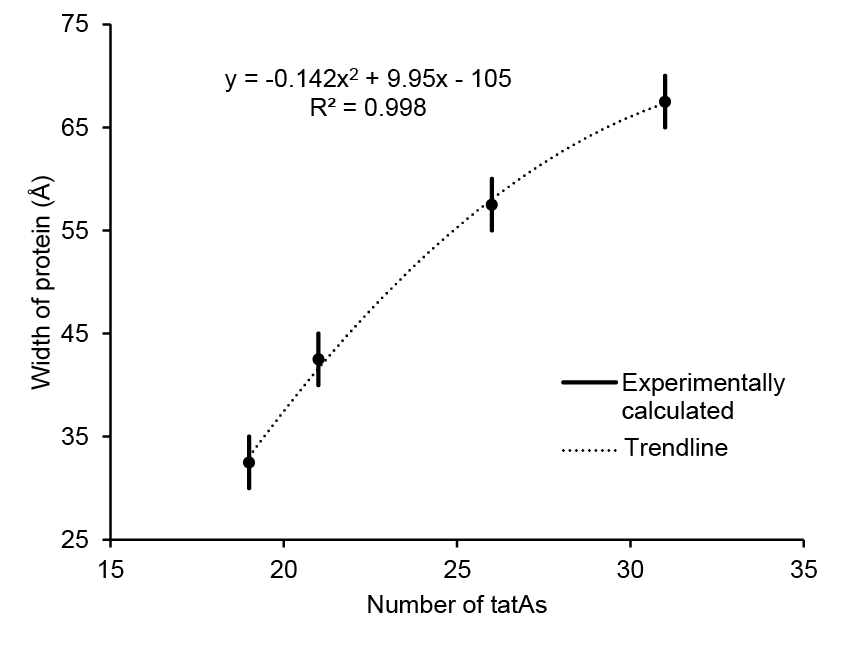
**

**Figure S1 – Calculation of the number of TatA proteins required for each translocated protein**

Using data from [1], the diameter of the channel formed by TatA proteins was plotted to determine how many TatAs are required to transport a Tat-translocated protein. The estimated width of each Tat-translocated protein was calculated from their molecular weight, assuming a spherical shape [2]. The resulting value was plugged into the trendline equation. The number of required TatA proteins was rounded up to the nearest integer and inserted into the Tat-pathway template reactions (Additional file 3).


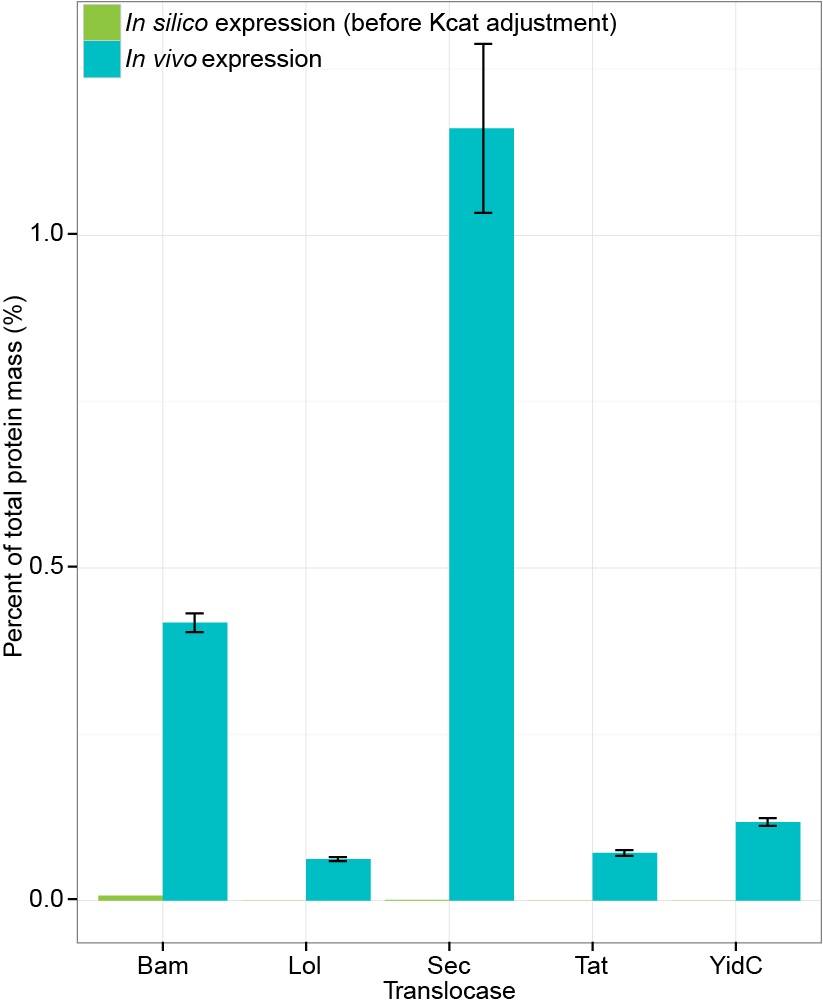


**Figure S2 - *In silico* protein expression of translocase pathways before the addition of enzyme turnover rates**

A bar graph showing simulation results (green) of translocase pathway protein levels from *i*JL1678-ME without translocase turnover rates and measured *in vivo* expression levels (blue) using RNA-seq as a proxy for protein production (R^2^=0.047, p-val=0.73). Results were taken from glucose M9 minimal media conditions.


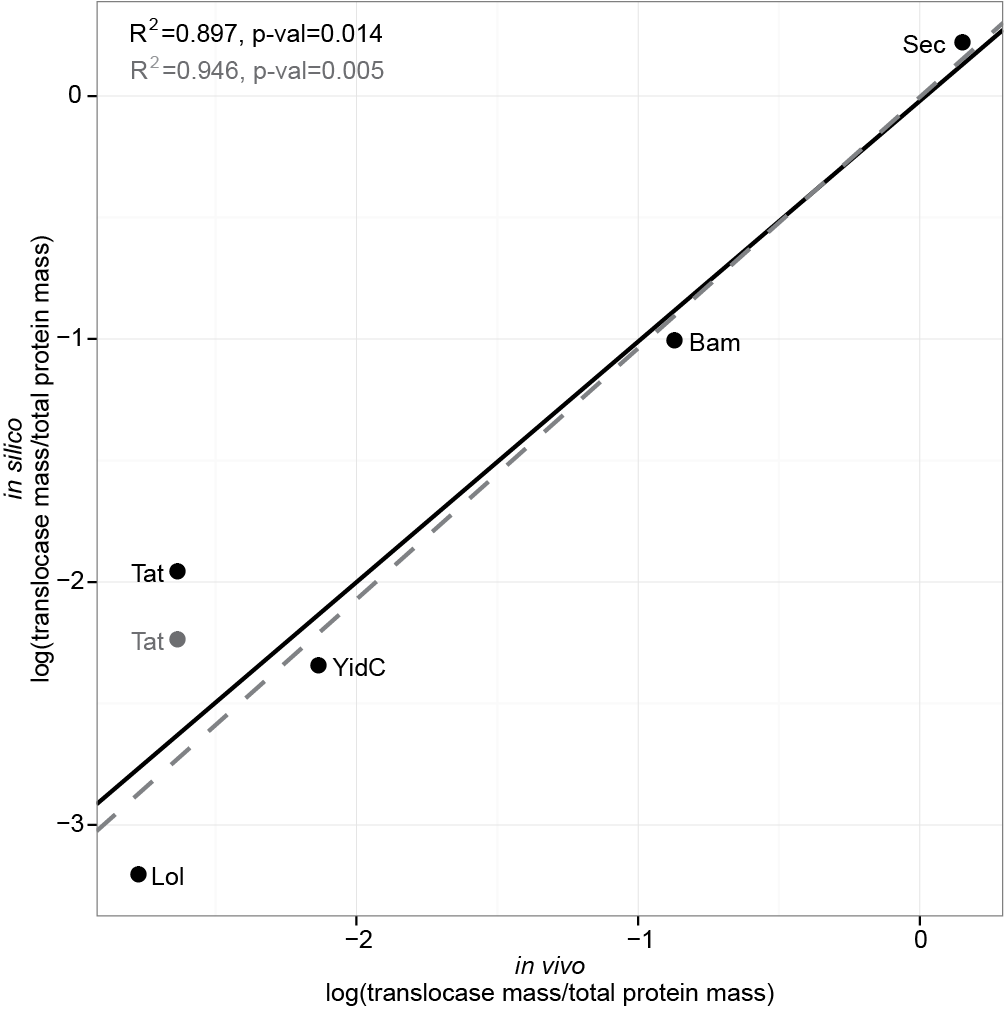


**Figure S3 – Comparison of *in silico* verses *in vivo* protein expression of translocase pathways**

Shown is a scatterplot comparing *in silico* and *in vivo* translocase protein levels. Gray represents new calculations when the mass of TatBC is lowered four-fold to account for TatBC’s ability to simultaneously translocate multiple substrates.


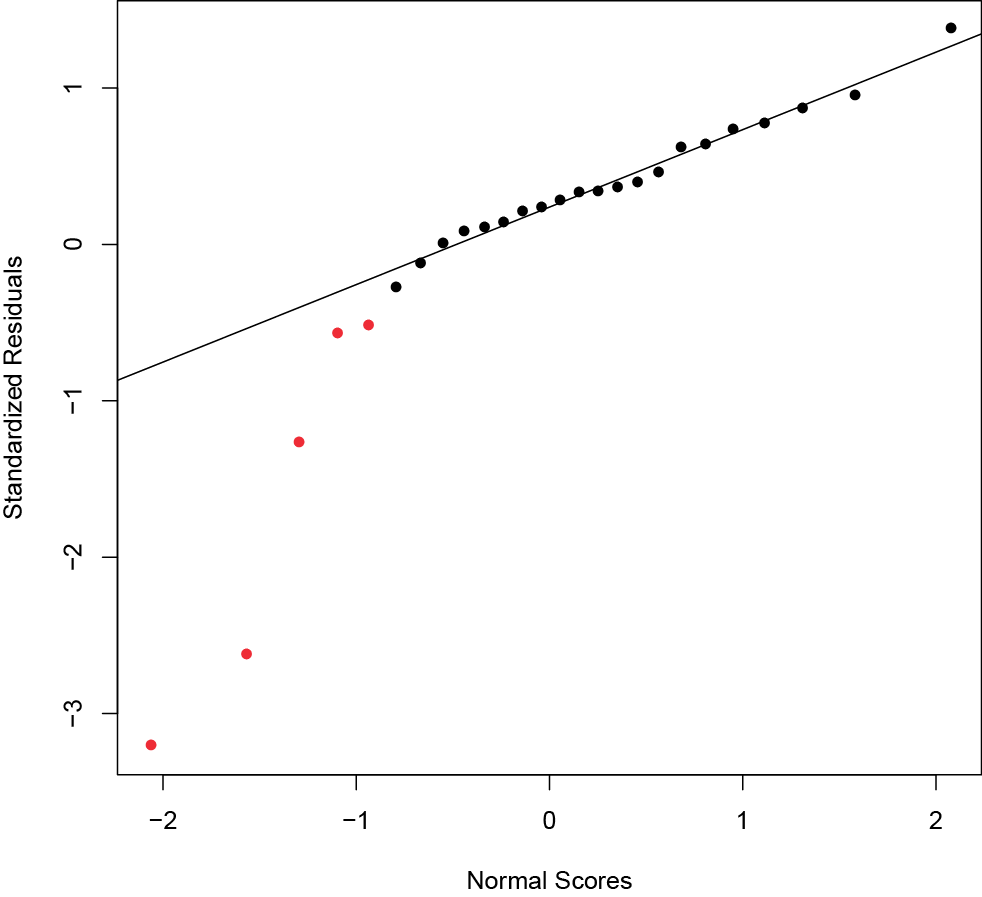


**Figure S4 - Additional data for the linear model analysis**

Points represent predicted (*in silico*) versus measured (*in vivo*) protein masses categorized by function and compartment for the proteins which were reconstructed in *i*JL1678-ME. The normal probability plot of rankit scores against standardized residuals of a linear regression over all data points demonstrates that several points (red) do not fall within a normal distribution.

**
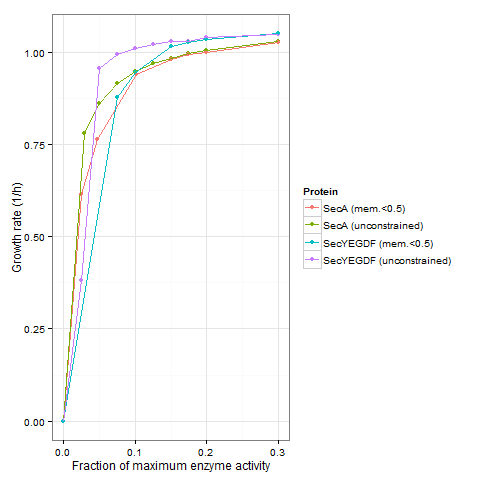
**

**Figure S5 – The effects of limiting the Sec pathway with membrane limitations**

Shown is a plot comparing SecA and SecYEGDF inhibition while the membrane is and is not constrained to 0.5. The membrane constraint affects overall growth rate at very low enzymatic levels (<0.2). Ribosome inhibition is not shown since the membrane constraint does not affect simulation results.

**References**

1. Gohlke U, Pullan L, McDevitt CA, Porcelli I, Leeuw E de, Palmer T, Saibil HR, Berks BC: **The TatA component of the twin-arginine protein transport system forms channel complexes of variable diameter**. *Proc Natl Acad Sci U S A* 2005, **102**:10482–10486.

2. Harpaz Y, Gerstein M, Chothia C: **Volume changes on protein folding**. *Structure* 1994, **2**:641–649.
